# The *FGF14* GAA repeat expansion is a major cause of ataxia in the Cypriot population

**DOI:** 10.1093/braincomms/fcae479

**Published:** 2025-01-03

**Authors:** Ioannis Livanos, Christina Votsi, Kyriaki Michailidou, David Pellerin, Bernard Brais, Stephan Zuchner, Marios Pantzaris, Kleopas A Kleopa, Eleni Zamba Papanicolaou, Kyproula Christodoulou

**Affiliations:** Neurogenetics Department, The Cyprus Institute of Neurology and Genetics, Nicosia 2371, Cyprus; The Cyprus Institute of Neurology and Genetics is a member of the European Reference Network-Rare Neurological Diseases (ERN-RND), Research Management Unit, Institute of Medical Genetics and Applied Genomics, University Hospital Tübingen, Tübingen 72076, Germany; Neurogenetics Department, The Cyprus Institute of Neurology and Genetics, Nicosia 2371, Cyprus; The Cyprus Institute of Neurology and Genetics is a member of the European Reference Network-Rare Neurological Diseases (ERN-RND), Research Management Unit, Institute of Medical Genetics and Applied Genomics, University Hospital Tübingen, Tübingen 72076, Germany; The Cyprus Institute of Neurology and Genetics is a member of the European Reference Network-Rare Neurological Diseases (ERN-RND), Research Management Unit, Institute of Medical Genetics and Applied Genomics, University Hospital Tübingen, Tübingen 72076, Germany; Biostatistics Unit, The Cyprus Institute of Neurology and Genetics, Nicosia 2371, Cyprus; Dr. John T. Macdonald Foundation Department of Human Genetics and John P. Hussman Institute for Human Genomics, University of Miami Miller School of Medicine, Miami, FL 33136, USA; Department of Neurology and Neurosurgery, Montreal Neurological Hospital and Institute, McGill University, Montreal, QC, CanadaH3A 2B4; Department of Neuromuscular Diseases, UCL Queen Square Institute of Neurology and The National Hospital for Neurology and Neurosurgery, University College London, London WC1N 3BG, UK; Department of Neurology and Neurosurgery, Montreal Neurological Hospital and Institute, McGill University, Montreal, QC, CanadaH3A 2B4; Dr. John T. Macdonald Foundation Department of Human Genetics and John P. Hussman Institute for Human Genomics, University of Miami Miller School of Medicine, Miami, FL 33136, USA; The Cyprus Institute of Neurology and Genetics is a member of the European Reference Network-Rare Neurological Diseases (ERN-RND), Research Management Unit, Institute of Medical Genetics and Applied Genomics, University Hospital Tübingen, Tübingen 72076, Germany; Neuroimmunology Department, The Cyprus Institute of Neurology and Genetics, Nicosia 2371, Cyprus; The Cyprus Institute of Neurology and Genetics is a member of the European Reference Network-Rare Neurological Diseases (ERN-RND), Research Management Unit, Institute of Medical Genetics and Applied Genomics, University Hospital Tübingen, Tübingen 72076, Germany; Neuroscience Department, The Cyprus Institute of Neurology and Genetics, Nicosia 2371, Cyprus; Centre for Neuromuscular Disorders, The Cyprus Institute of Neurology and Genetics, Nicosia 2371, Cyprus; The Cyprus Institute of Neurology and Genetics is a member of the European Reference Network-Rare Neurological Diseases (ERN-RND), Research Management Unit, Institute of Medical Genetics and Applied Genomics, University Hospital Tübingen, Tübingen 72076, Germany; Centre for Neuromuscular Disorders, The Cyprus Institute of Neurology and Genetics, Nicosia 2371, Cyprus; Neuroepidemiology Department, The Cyprus Institute of Neurology and Genetics, Nicosia 2371, Cyprus; Neurogenetics Department, The Cyprus Institute of Neurology and Genetics, Nicosia 2371, Cyprus; The Cyprus Institute of Neurology and Genetics is a member of the European Reference Network-Rare Neurological Diseases (ERN-RND), Research Management Unit, Institute of Medical Genetics and Applied Genomics, University Hospital Tübingen, Tübingen 72076, Germany

**Keywords:** *FGF14*, spinocerebellar ataxia 27B, SCA27B, autosomal dominant cerebellar ataxia, Cyprus

## Abstract

Dominantly inherited intronic GAA repeat expansions in the fibroblast growth factor 14 gene have recently been shown to cause spinocerebellar ataxia 27B. Currently, the pathogenic threshold of (GAA)_≥300_ repeat units is considered highly penetrant, while (GAA)_250–299_ is likely pathogenic with reduced penetrance. This study investigated the frequency of the GAA repeat expansion and the phenotypic profile in a Cypriot cohort with unresolved late-onset cerebellar ataxia. We analysed this trinucleotide repeat in 155 patients with late-onset cerebellar ataxia and 227 non-neurological disease controls. The repeat locus was examined by long-range PCR followed by fragment analysis using capillary electrophoresis, agarose gel electrophoresis and automated electrophoresis. A comprehensive comparison of all three electrophoresis techniques was conducted. Additionally, bidirectional repeat-primed PCRs and Sanger sequencing were carried out to confirm the absence of any interruptions or non-GAA motifs in the expanded alleles. The (GAA)_≥250_ repeat expansion was present in 12 (7.7%) patients. The average age at disease onset was 60 ± 13.5 years. The earliest age of onset was observed in a patient with a (GAA)_287_ repeat expansion, with ataxia symptoms appearing at 25 years of age. All patients with spinocerebellar ataxia 27B displayed symptoms of gait and appendicular ataxia. Nystagmus was observed in 41.7% of the patients, while 58.3% exhibited dysarthria. Our findings indicate that spinocerebellar ataxia 27B represents the predominant aetiology of autosomal dominant cerebellar ataxia in the Cypriot population, as this is the first dominant repeat expansion ataxia type detected in this population. Given our results and existing research, we propose including fibroblast growth factor 14 GAA repeat expansion testing as a first-tier genetic diagnostic approach for patients with late-onset cerebellar ataxia.

## Teaser

The GAA repeat expansions in the FGF14 gene cause SCA27B. Livanos *et al*. found that 7.7% of unresolved ataxia Cypriot patients had the GAA repeat expansion. The primary clinical symptoms included gait, appendicular ataxia, nystagmus, and dysarthria. SCA27B is now considered one of the predominant causes of ataxia in Cyprus.

## Graphical Abstract

**Graphical Abstract:**
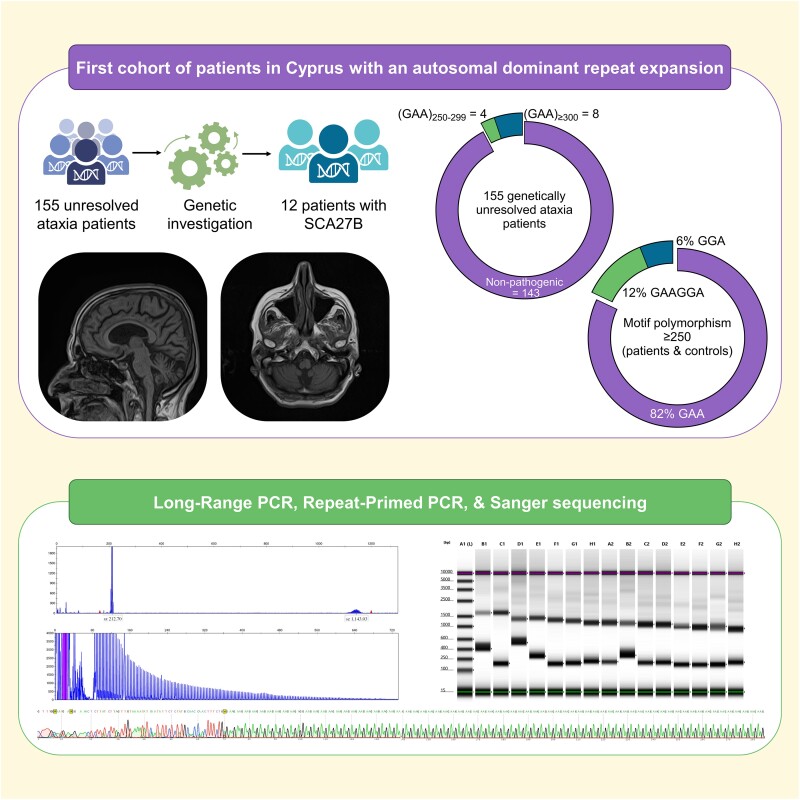

## Introduction

Spinocerebellar ataxia 27B (SCA27B) (MIM: 620174) is a dominantly inherited neurodegenerative disease caused by (GAA)•(TTC) trinucleotide repeat expansion in the first intron of isoform 1b of the fibroblast growth factor 14 (*FGF14*) gene.^1,2^ This pathogenic repeat expansion has emerged as one of the most common causes of late-onset cerebellar ataxia (LOCA).^1,3^ The primary clinical characteristics of SCA27B encompass slowly progressive cerebellar ataxia, episodic symptoms, ocular motor signs, such as downbeat nystagmus and visual disturbances.^1,2,4,5^

The *FGF14*-1b isoform encodes a 252-amino-acid protein localized to the cytoplasm and is primarily expressed in the adult brain, especially the cerebellum.^6,7^ It interacts with and modulates the function of voltage-gated sodium (Na_V_) channels, aiding their localization to the axon initial segment. FGF14 regulates channel gating and axonal targeting.^8,9^ It is essential for spontaneous and repetitive rhythmic firing of cerebellar Purkinje neurons, vital for balance and motor control.^10^

Currently, the pathogenic threshold of (GAA)_≥300_ repeat units is disease-causing and highly penetrant, while expansions of (GAA)_250–299_ repeat units are considered likely pathogenic with reduced penetrance.^1,2,11^ The expansion is frequently referred to as a GAA repeat expansion. However, due to the gene being located on the minus strand of the genome, the repeat expansion consists of TTC nucleotides in the gene context. Previous reports observed that interrupted or non-GAA-pure repeats in the *FGF14* repeat locus are not associated with SCA27B.^1,12,13^

Many diverse cohorts presenting with LOCA have been studied.^1-3,14-24^ SCA27B is rapidly gaining global recognition as one of the most prevalent forms of autosomal dominant cerebellar ataxias (ADCAs) in people of European descent, with a similar frequency as the common polyglutamine SCA subtypes.^5,15^ Cyprus is situated in the Eastern Mediterranean Sea, and contrary to other European populations, no ADCA cases have been previously described in this population. Therefore, we wanted to investigate the recently identified repeat expansion and determine its existence in Cypriot LOCA patients. The Cypriot population belongs to a genetic continuum extending from Southern Italy to Lebanon, through the Greek islands.^25^ This report presents the genetic and clinical findings from our investigation into *FGF14* GAA repeat expansion in a Cypriot ataxia cohort. This study includes 155 patients with unsolved LOCA and 229 non-neurological disease controls. Here, we report the first SCA occurrence in the Cypriot population. Our findings suggest that SCA27B is the predominant aetiology of ADCA in Cyprus thus far, representing the first dominant repeat expansion ataxia to be identified in this population. Testing for the *FGF14* GAA repeat expansion should be considered as a primary genetic diagnostic approach for all undiagnosed patients with LOCA.

## Materials and methods

### Study design

A total of 155 unrelated index patients who presented with LOCA were filtered from the in-house database and were included in this study, along with 229 non-neurological disease controls. Patients were eligible for inclusion if they had developed ataxia at or after the age of 20 years and tested negative for the SCA1, SCA2, SCA3, SCA6, SCA7, SCA8, SCA10, SCA12, SCA36, DRPLA, Friedreich’s ataxia (FRDA) and the pathogenic *RFC1* repeat expansions.^26,27^ Additionally, based on the phenotypic features of each patient, other ataxia-causative recessive variants identified in Cypriot families were previously tested and excluded.

### Clinical data collection

The Cyprus Institute of Neurology and Genetics has been overseeing the care of ataxia patients through on-site clinicians for many years. The clinical data included in our study were retrospectively collected.

### *FGF14* targeted analysis

A multi-step screening process was followed for the *FGF14* GAA repeat expansion investigation. This approach closely parallels the previously proposed stepwise methodology.^11^ As described below, some modifications were introduced to the reported strategy to minimize the use of Sanger or long-read sequencing (LRS) and to get the most accurate allele sizing even for alleles beyond the capillary electrophoresis detection limit.

1. The *FGF14* repeat locus was interrogated using fluorescent long-range PCR (LR-PCR) amplification and fragment analysis by capillary electrophoresis. Amplicons were analysed using the ABI 3500xl Genetic Analyser and the GeneMapper software version 6.0 [Applied Biosystems (ABI), Waltham, MA, USA].
2. After the initial analysis, samples with a single allele were further amplified using unlabelled LR-PCR and analysed by TapeStation 4150 automated electrophoresis instrument (Agilent Technologies, Santa Clara, CA, USA). This was done to confirm homozygosity for non-expanded alleles or detect expanded alleles exceeding the detection limit of capillary electrophoresis (∼350 repeats). Moreover, all samples measured by capillary electrophoresis to contain ≥200 repeats were also analysed using LR-PCR, followed by automated electrophoresis. The re-examination of amplicons is necessary as capillary electrophoresis underestimates the size of repeat-containing expanded alleles.^11^ Amplicons were analysed using automated electrophoresis with the High Sensitivity D5000 ScreenTape assay following the manufacturer’s protocol (SD-UF0000090 Rev. B) and analysed using the TapeStation software version 5.1 (Agilent Technologies). Agarose gel electrophoresis was conducted for comparison of size estimates, despite its redundancy. The fluorescent LR-PCR products were electrophoresed for 1 h at 120 volts, in a 1.5% agarose gel.
3. To enhance the accuracy of the repeat size estimates, seven calibrator samples with known allele sizes were employed to adjust the automated electrophoresis repeat size estimates in patients and controls. The allele sizes were previously estimated through targeted LRS (Oxford Nanopore Technologies, Oxford, UK). Least squares regression was used to generate a regression model with a slope-intercept equation to adjust the automated electrophoresis measurements (Supplementary Fig. S1).
4. To confirm the uninterrupted motif of the GAA repeat expansion, bidirectional (GAA)•(TTC) repeat-primed PCR (RP-PCR) was performed using capillary electrophoresis as previously described.^2,11^ Based on the nanopore-adjusted automated electrophoresis measurements, this analysis was performed on samples with at least one allele of ≥250 repeat units. Cases observed with an interrupted profile or no sawtooth profile underwent further screening.
5. Sanger sequencing of expanded alleles in the forward and reverse direction was used in conjunction with the bidirectional RP-PCRs, (i) to further confirm the uninterrupted GAA repeat motif and (ii) to interrogate all samples presenting with an absent sawtooth profile for the GAA repeat expansion, thereby enabling the detection of other motifs. Sanger sequencing was performed using the BigDye Terminator v1.1 Cycle Sequencing Kit and the ABI 3500xl Genetic Analyser. The electropherograms were visualized using the Sequencing Analysis software version 6.0 (ABI).
6. Bidirectional (GAAGGA)•(TCCTTC) RP-PCR was developed to interrogate the non-pathogenic GAAGGA repeat expansion^1^ to minimize the future need to employ Sanger sequencing. A multiplex design of distinctly labelled primers (Supplementary Fig. S2 and Table S1) targeting the GAA and the GAAGGA motifs enabled the combined fragment analysis of two RP-PCR reactions in each direction (5′ and 3′) in a single capillary, thus producing a single electropherogram. All samples confirmed by Sanger sequencing to have the GAAGGA motif were also analysed using this assay.

### Statistical analysis

Bland–Altman analyses were used to compare the measurements of the same expanded allele and assess method agreement.^28^ Electrophoresis techniques were compared with nanopore-adjusted automated electrophoresis measurements using Passing–Bablok regression.^29^ Simple linear regression analysis was performed to explore the relationship between allele size and age of onset and the coefficient of determination (*R*^2^) was calculated. A *P*-value of <0.05 was considered statistically significant. All statistical analyses were conducted using GraphPad Prism software version 10 (GraphPad Software, San Diego, CA, USA).

### Patient consent

Written informed consent was obtained from all control individuals participating in the study. For the patients, no additional informed consent was requested as the test was in line with the original request for diagnostic testing. The study was conducted according to the guidelines of the Declaration of Helsinki.

### Ethical approval

This study has been approved by the National Bioethics Committee of Cyprus (ΕΕΒΚ/ΕΠ/2013/28). The study was conducted in compliance with the General Data Protection Regulation of the European Union which pertains to the safeguarding of individuals, regarding the processing of personal data (EU 2016/679).

## Results

### *FGF14* GAA repeat expansion sizing and technique comparison

**Figure 1:**
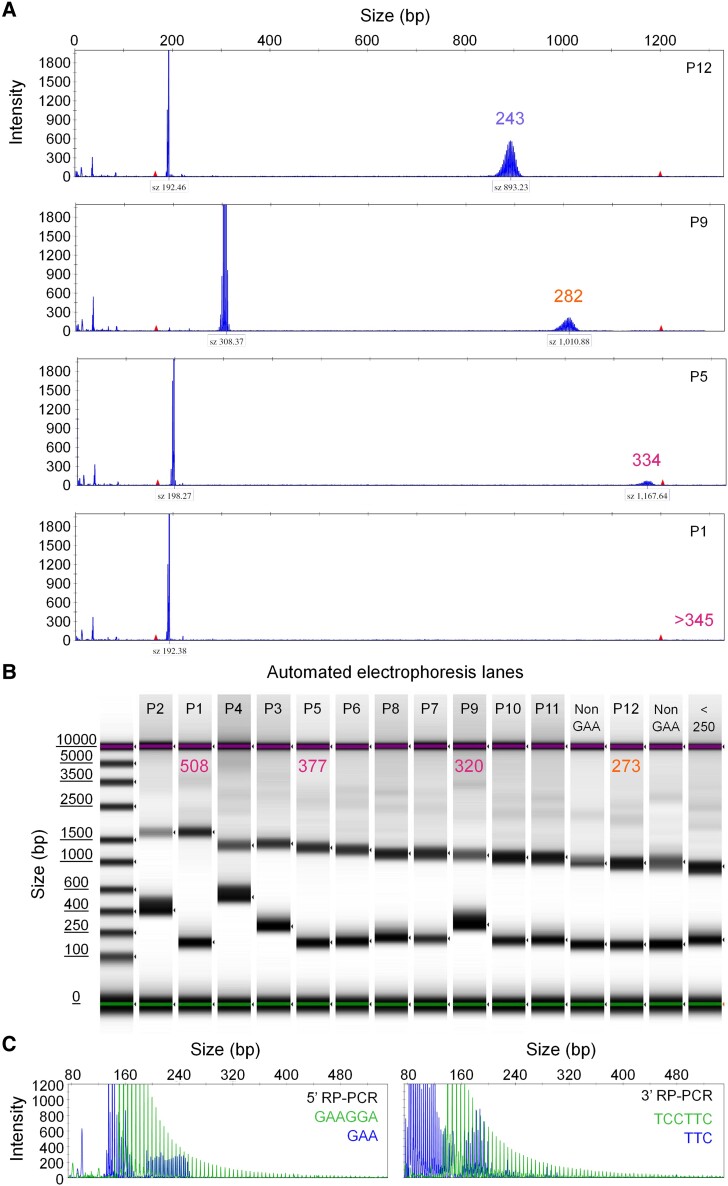
**Molecular analysis of the GAA repeat locus.** The repeat size estimation of the expanded allele generated by (**A**) ABI 3500xl 50 cm capillary electrophoresis and (**B**) TapeStation 4150 automated electrophoresis (before nanopore adjustment), is indicated for each of the same four patients. The repeat sizes were determined by converting the base pair (bp) measurements from the electrophoresis instruments into trinucleotide repeat units. (**C**) GAA and GAAGGA bidirectional RP-PCRs target the 5′ and the 3′ *FGF14* repeat locus of an index patient carrying 40 and 291 trinucleotide repeat units. The expanded allele contains GAAGGA repeat units hence SCA27B is excluded from this index patient. The GAA repeats overlapping the GAAGGA repeats, correspond to the smaller allele.

We screened all study participants to determine the percentage of expanded alleles in our cohort. The repeat size of the expanded alleles was initially evaluated using fragment analysis. Bidirectional RP-PCRs and Sanger sequencing confirmed the pathogenic repeat motif in relevant cases. However, previous studies reported that capillary electrophoresis consistently underestimated the size of the expanded alleles compared with targeted LRS and gel electrophoresis.^11,18^ Moreover, fragment analysis with a 50-cm capillary can effectively measure up to 1200 bp (∼350 repeats). Therefore, accurate sizing could not be obtained on samples above this threshold. Four representative samples sized by capillary and automated electrophoresis (before adjustment) are shown in Fig. 1A and B. Given the above limitations and due to the unavailability of LRS in our institute we utilized the Tapestation automated electrophoresis instrument to analyse the expanded alleles.

**Figure 2:**
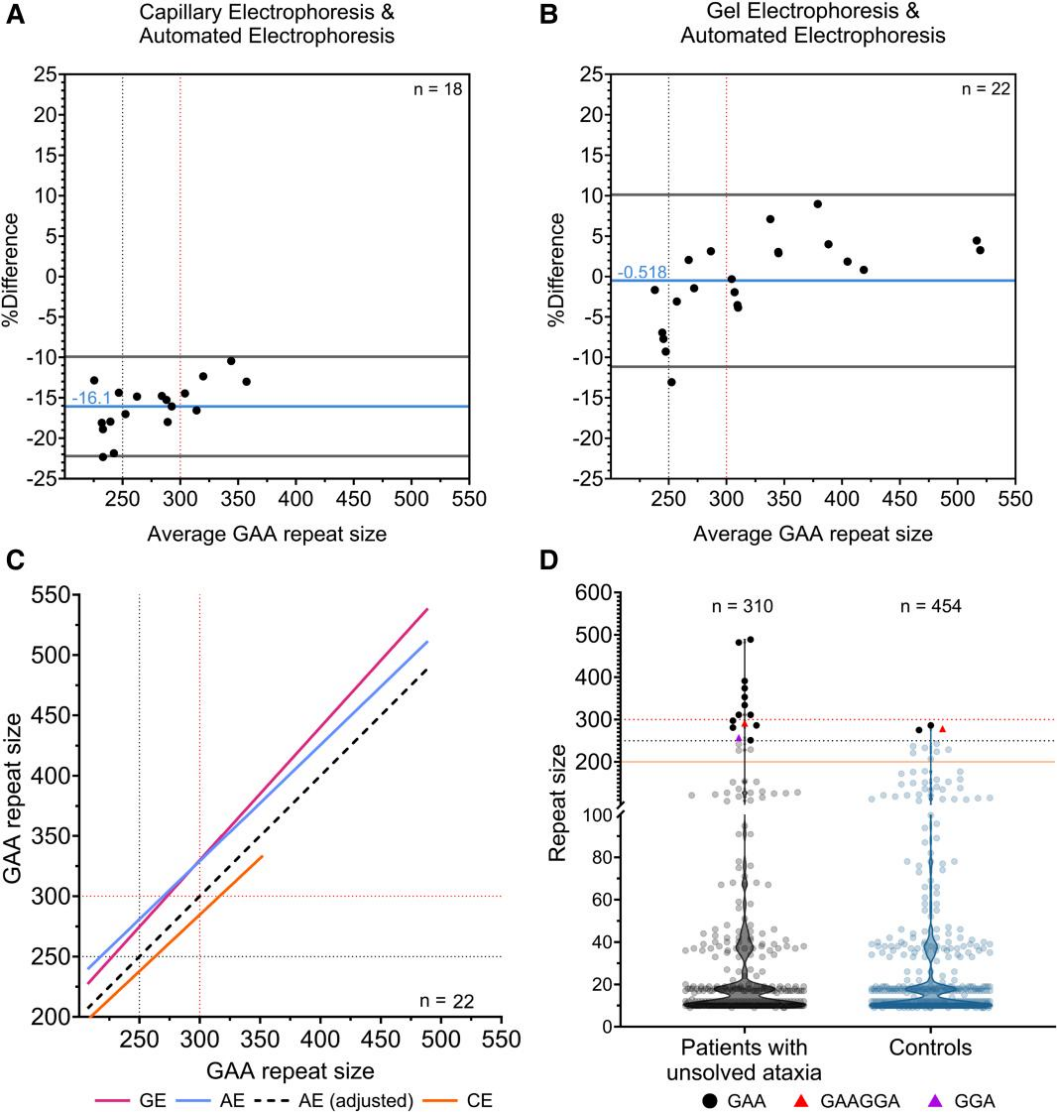
**GAA expansion size estimation using three electrophoresis techniques.** Bland–Altman plots illustrate the percentage difference between size estimates by (**A**) automated and capillary electrophoresis or (**B**) automated and agarose gel electrophoresis. This percentage difference is expressed as the average of the two measurements for each sample. Each data point represents an individual (patient or control) with a pure GAA repeat expansion. (**C**) Passing–Bablok regression for the expanded allele sizes was measured using different electrophoresis techniques. (**D**) The allele distribution of the *FGF14* GAA locus for both chromosomes of 155 index ataxia patients and 227 controls, shown as violin plots.The upper and middle dotted lines denote the pathogenic and reduced penetrance thresholds of 300 and 250 repeat units, respectively. CE (capillary electrophoresis ABI 3500xl 50-cm), AE (automated electrophoresis TapeStation 4150) and GE (agarose gel electrophoresis 1.5%). The lower solid line represents a technical threshold; below this line, allele sizing is based on capillary electrophoresis, while above the line, allele sizing is based on nanopore-adjusted automated electrophoresis readings.

Specifically, samples with expanded alleles estimated to contain ≥200 repeat units (measured by capillary electrophoresis), were subjected to further testing using gel and automated electrophoresis. Both methods can effectively determine the repeat size of any expanded allele >350 repeats (Fig. 1B, only TapeStation measurements shown). A comparison between capillary and automated electrophoresis revealed a significant discrepancy in the size estimation of expanded alleles (Fig. 2A). A comprehensive comparison of three electrophoresis techniques was conducted with 22 samples carrying a (GAA)_≥200_ repeat expansion. The Bland–Altman analysis to assess agreement between two methods,^28^ identified a negative mean bias of −16.1% [−9.9% to −22.2%, 95% limits of agreement (LoA)] between capillary and automated electrophoresis and an insignificant negative mean bias of −0.5% (10.1% to −11.2%, 95% LoA) was observed between agarose gel and automated electrophoresis (Fig. 2A and B).

Automated and gel electrophoresis have demonstrated a high degree of similarity in estimating repeat allele sizes (Fig. 2B). To ensure repeat size estimations generated by automated electrophoresis are as close as possible to the true repeat size, these were adjusted using seven calibrators containing expanded alleles, previously measured by targeted LRS and a linear relationship was established. A Passing–Bablok regression model (Fig. 2C) was generated by comparing the size of LR-PCR products of expanded alleles.^29^ As a result of the above approaches, we identified 12 patients with cerebellar ataxia (12/155, 7.7%) carrying an *FGF14* (GAA)_≥250_ repeat expansion.

### *FGF14* GAA repeat expansion prevalence

As mentioned above, we studied the allelic distribution of the *FGF14* repeat locus with three electrophoresis methods, in 155 patients with unresolved LOCA and 227 non-neurological disease controls. In this cohort, eight (5.1%) patients carried a pathogenic (GAA)_≥300_ repeat expansion, whereas four (2.6%) patients had a reduced penetrance (GAA)_250–299_ expansion. The median repeat count of patients with (GAA)_≥250_ repeat expansion was 323 repeat units (range, 251–489 repeats). Two (0.9%) non-neurological disease controls were identified with a (GAA)_286_ and a (GAA)_275_ expansion. No controls were identified carrying the (GAA)_≥300_ expansion (Fig. 2D). The (GAA)_10_ was the most prevalent repeat allele, representing 31% (96/310) and 34% (156/454) of all alleles in the genetically unresolved ataxia and control cohorts, respectively. In the ataxia cohort, the median repeat number was 17 [interquartile range (IQR), 10–37] trinucleotide repeat units, while for the non-disease controls, it was 12 (IQR, 10–33) repeat units (Fig. 2D). The smallest allele identified in both groups was nine repeats.

Sanger sequencing analysis of the *FGF14* repeat locus in both index patients and controls with an expanded allele of ≥250 repeat units revealed the presence of non-GAA-pure repeat expansions. Consistent with prior reports with participants of European descent,^1^ the GAAGGA hexanucleotide motif emerged as the most frequent non-(GAA)_≥250_ expanded allele. Among all the expanded alleles surpassing the reduced penetrance threshold from both cohorts, 82% (14/17, 12 patients; 2 controls) consisted of pure GAA units, 12% (2/17, 1 index patient; 1 control) of GAAGGA units, and 6% (1/17, 1 index patient) of pure GGA units. Other reported motifs, such as the [(GAA)_m_(GCA)*_n_*]_z,_ were not detected in our cohort.

Given the challenges associated with sequencing large repeat motifs, we designed a bidirectional (GAAGGA)•(TCCTTC) RP-PCR to be used for the future screening of this common repeat. This second approach unambiguously confirmed the presence of GAAGGA repeats. Distinct labelling of the primers also enabled electrophoresis of the two RP-PCRs in a single capillary. Representative results of an index patient are shown in Fig. 1C.

### Clinical characteristics of SCA27B patients

**Figure 3:**
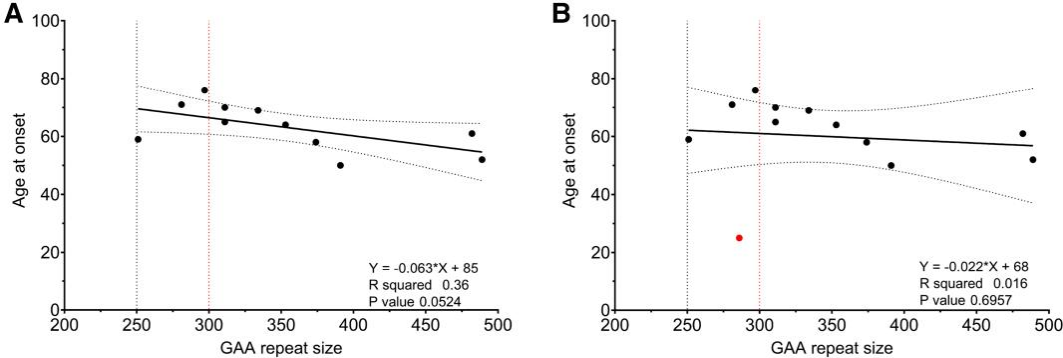
**Age at onset correlation.** A simple linear regression analysis was conducted to investigate the association between allele size and age of onset. The scatter plot illustrates the direction and the strength of the correlation between the repeat size (*x*-axis) and the age at disease onset (*y*-axis). The SCA27B patients are plotted with (**A**) or without the outlier (**B**). No significant correlation is identified between the age of onset and the number of repeat units (*P* > 0.05). Each data point represents a SCA27B patient.

The *FGF14* (GAA)_≥250_ repeat expansion was detected equally in both males and females. The mean age at disease onset was 60.0 ± 13.5 years and the mean age at first examination was 66.3 ± 14.4 years. There was no association between GAA size and age of onset (Fig. 3A). One patient with a (GAA)_286_ repeat expansion had an age of onset at 25 years and was diagnosed with ataxia at the age of 28. Upon exclusion of this outlier from the analysis (Supplementary Table S2), a weak inverse correlation between repeat size and age of onset began to emerge. However, this correlation did not reach statistical significance (Fig. 3B).

**Figure 4:**
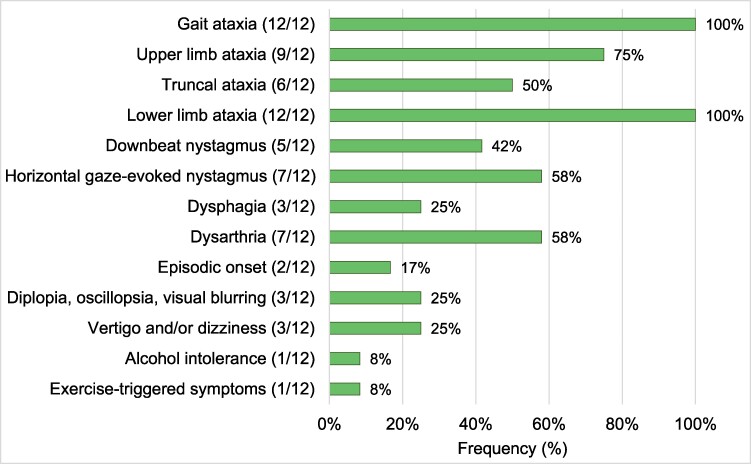
**Main clinical features of SCA27B patients.** Frequency of SCA27B-related phenotypic features in 12 patients diagnosed with (GAA)_≥250_. Numbers in brackets indicate the number of affected patients, with the specific phenotypic feature over the total number of patients assessed for this feature.

All patients in the *FGF14*-GAA-positive cohort were diagnosed with gait and appendicular ataxia. Downbeat nystagmus and dysarthria were observed in 5 (41.7%) and 7 (58.3%) out of 12 patients, respectively. Dysphagia and visual disturbances such as diplopia, oscillopsia or visual blurring were present in 3/12 (25%). Episodic symptoms were observed in only 2/12 (16.7%) patients (Fig. 4). Specifically, these two patients developed episodic symptoms at the age of 52 and 58, and permanent ataxia at the age of 55 and 67, respectively. Interestingly, one patient had a history of heavy smoking. However, following the onset of ataxia, this patient developed an intolerance to smoking tobacco.

**Figure 5:**
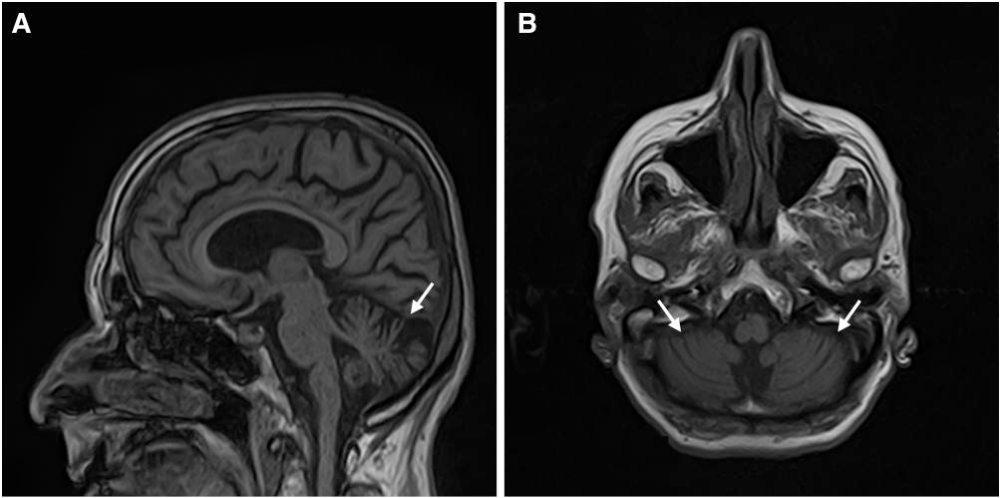
**Brain MRI findings of a patient with the *FGF14* GAA repeat expansion.** Moderate cerebellar atrophy (white arrows) in a female patient (P11) with SCA27B, (**A**) in sagittal T1 and (**B**) axial T1 views.

Nerve conduction studies (NCSs) were performed in five patients. One (20%) patient had length-dependent sensorimotor axonal polyneuropathy. Magnetic resonance imaging (MRI) was obtained for all patients and cerebellar atrophy was observed in five (5/12, 41.7%) patients. The cerebellar atrophy was limited to the vermis in three patients (3/12, 25%) (Fig. 5A and B) and extended to the hemispheres in another two (2/12, 12.5%) patients. Global atrophy was present in one (1/12, 8.3%) patient and white matter lesions (WMLs) were detected in four (4/12, 33.3%) patients. In three patients, no abnormalities were found. The detailed clinical presentation of the patients with a (GAA)_≥250_ repeat expansion is presented in Supplementary Table S2.

## Discussion

This study explored the frequency and phenotypic spectrum of SCA27B within the Republic of Cyprus. We screened a cohort of 155 genetically unresolved LOCA patients and 229 non-neurological disease controls. We identified 12 patients (12/155, 7.7%) with the *FGF14* (GAA)_≥250_ repeat expansion. Eleven patients have a Cypriot origin and one is Russian (Supplementary Table S2). Specifically, the *FGF14* (GAA)_≥300_ was present in eight (5.1%) patients and the (GAA)_250–299_ repeat expansion was in four (2.6%) patients. Additionally, two controls (0.9%) had reduced penetrance (GAA)_250–299_ repeat expansion. This corroborates previous studies in European populations, where the prevalence of the reduced penetrance allele was 0.6–1.7%, in controls.^1,2,16^ According to the literature, the relationship between expansion size and age of onset remains inconclusive. This study also found no significant correlation between GAA size and age of onset. Approximately half of the previous studies reported no significant correlation,^16-20^ while the remaining studies identified a weak inverse correlation between the repeat expansion size and age at disease onset.^1,2,11,14^

Earlier studies screened patients for GAA expansion with an age of onset ≥30 years.^1,3,11^ Follow-up reports identified individuals in their twenties who exhibited symptoms of ataxia and tested positive for SCA27B.^15,16,21^ As a result, this study considered individuals with unresolved ataxia with an age of onset of at least 20 years as eligible participants. The mean age of disease onset and age at first examination was 60.0 and 66.3, respectively. Two phenotypically normal controls with an age of 67 at the time of collection were found to carry a (GAA)_250–299_ expansion. Although this finding does not rule out the possibility of future disease manifestation since some patients may manifest their first symptoms after age 80, SCA27B typically presents between 55 and 67.^1,5^ No controls were identified with the fully penetrant pathogenic repeat expansion.

Here, the reported SCA27B frequency is lower than previously published rates in other European populations, where the reported rate among patients with unsolved LOCA is between 15 and 30%.^5^ The highest frequency was reported in French Canadians at 61%, indicating a possible founder effect in that population.^1^ Specifically, in European populations, the frequency was 28% in Spain, 31% in Germany, 11.8% in France and 5.4% in Serbia.^3,15,16,23^ The frequency of SCA27B was compared with other SCAs in a German cohort of genetically determined patients. SCA27B comprised 16% of all SCA index patients representing the second most common SCA, after SCA3 with 19%.^15^ The first study on the US population with undiagnosed ataxia of predominantly White individuals, recruited from five academic centres, identified 55 out of 732 patients (7.5%) with the GAA repeat expansion.^24^ The prevalence in Brazil is 9%, while the prevalence among South-Asian and East-Asian populations is significantly lower, as evidenced by rates observed in India (1.8%), China (1.3%) and Japan (1.2%).^18,19,21,30^

The discrepancy in frequency rate between the Cypriot population and the other European populations could be attributed to the unique geographical context of Cyprus, as it is a small island in the eastern Mediterranean Sea. The Cypriot population occupies an intermediary position within a genetic spectrum extending from the Levant (also known politically as the Middle East) to Southeast Europe. Based on the Y-chromosome analysis, the Cypriot patrilineage predominantly traces its origins to Calabrian-Italian, Lebanese and Cretan-Greek populations.^25^ These intricate genetic dynamics underscore the complex interplay of regional isolation, historical migrations and population admixture in shaping the prevalence of SCA27B in this population. A study on Greek patients with LOCA, a population that shares substantial genetic similarities with the Cypriot population, identified the frequency of repeat expansion at 11.9% (19/160).^20^ Similarly, the prevalence of SCA27B in a large Italian cohort with unsolved LOCA patients was 13.4% (53/396).^22^ To our knowledge, no studies have been conducted on independent cohorts of Lebanese origin.

The phenotype of SCA27B patients mostly aligns with previous reports. Gait and appendicular ataxia were present in all patients. The MRI findings of this study align with previous findings, highlighting that cerebellar atrophy in SCA27B predominantly affects the vermis, consistent with the prominent clinical presentation of gait and midline ataxia, with less involvement of the hemispheres.^4,11,17,18,21^ Additionally, four patients with WMLs were also identified. In a recent study, it has been shown that SCA27B causes more than cerebellar atrophy. Specifically, different patterns of white matter abnormalities have been described by Chen *et al*.^31^ mainly involving the superior cerebellar peduncle.

This study also reports the identification of non-GAA-pure repeat expansions in the *FGF14* gene, similar to previous studies.^1,2,11,32^ Alternative repeat expansion motifs are not associated with SCA27B.^1,12,13^ In FRDA (MIM: 229300) alternative repeat expansions are also not associated with the disease. FRDA is an autosomal recessive disease caused by an intronic repeat expansion in the frataxin gene (*FXN*) and is the only other ataxia type caused by a GAA repeat expansion.^33^ However, the expansion is biallelic in FRDA. The GAA repeat expansion within the *FXN* gene leads to the formation of non-B DNA structures, inhibiting *FXN* transcription. Specifically, a purine-purine-pyrimidine (R•R•Y) intramolecular triplex DNA (H-DNA) and/or an intermolecular structure, termed ‘sticky’ DNA are formed.^34-36^ Non-GAA repeat expansions in the *FXN*, specifically GGA and GAAGGA expansions, are not associated with FRDA, as they do not form DNA secondary structures, thereby mitigating transcriptional inhibition.^37,38^ Furthermore, the greater the magnitude of interruptions, the greater the interference in forming secondary structures and the less the transcriptional inhibition.^37^ Even though the precise mechanism underlying the *FGF14* GAA repeat expansion remains elusive, perhaps a common repeat expansion molecular mechanism exists between SCA27B and FRDA. This conjecture arises from the observation that the same trinucleotide motif triggers both disorders. The GAA repeat expansion causes the formation of purine-rich *H-DNA by reverse-Hoogsteen hydrogen bonds,^34-37^ the formation of RNA/DNA hybrids (R-loops) that impede RNA polymerase II,^39^ or the transcriptional downregulation by epigenetic silencing.^40^

A common pathophysiological mechanism may underlie SCA27A (MIM: 193003) and SCA27B, as both diseases are caused by variants in the same gene. SCA27A is caused by heterozygous mutations in the *FGF14* gene, resulting in reduced expression and haploinsufficiency.^7^ SCA27B mechanism of disease causation is likely also the loss of function via haploinsufficiency. Future investigations could explore the molecular mechanism of how pure GAA repeat expansions lead to SCA27B and how interrupted expansions or other alternative motifs are not associated with the pathology.

The *FGF14* GAA repeat expansion is rapidly gaining global recognition as one of the most prevalent forms of LOCA,^1,2,5^ underscoring the need to establish a standardized diagnostic protocol in the absence of LRS, currently unavailable in many laboratories, to be implemented in the diagnostic setting.^11^ Our data indicate that TapeStation automated electrophoresis provides an alternative approach for genotyping expanded alleles. Our current diagnostic approach involves the utilization of capillary electrophoresis for the initial screening process and nanopore-adjusted automated electrophoresis to assess alleles containing ≥200 repeat units. While agarose gel electrophoresis remains a gold standard method, its semi-quantitative nature imposes certain limitations. Additionally, two bidirectional RP-PCRs and Sanger sequencing were employed. Capillary electrophoresis consistently yields smaller allele size estimates than those obtained using gel and automated electrophoresis. These findings corroborate previous findings demonstrating that capillary electrophoresis systematically underestimates the size of expanded alleles when compared with gel electrophoresis and targeted LRS.^11,18^ Repeat oligonucleotides tend to form secondary structures that have higher electrophoretic mobilities. This phenomenon occurs irrespective of the underlying genetic sequence. The faster migration rate observed in repeat-containing fragments can be attributed to altered electrophoretic properties of DNA enriched with repetitive sequences.^41-43^

This underestimation could result in false negative results and misdiagnosis, particularly for alleles near the reduced penetrance threshold. Automated and gel electrophoresis have no size limitations and can effectively measure large repeat alleles. In line with previous findings, we observed that gel electrophoresis tends to overestimate the repeat allele size compared with targeted LRS.^11^ This trend is also observed in automated electrophoresis, as both gel and automated electrophoresis generated similar repeat allele size estimates. Seven calibrators measured by targeted LRS were employed to adjust the allele size estimates generated by automated electrophoresis, increasing the accuracy of the large allele estimates. As a result, automated electrophoresis emerged as the preferred method for repeat allele sizing, offering a convenient approach for genotyping the *FGF14* repeat locus.

Resolving large repeat motifs is challenging with Sanger sequencing, with the most notable limitation being the sudden decrease in signal intensity. To reduce reliance on labour-intensive Sanger sequencing for the investigation of cases indicative of having alternative repeat expansion motifs, an RP-PCR for the common non-GAA motif (GAAGGA)•(TCCTTC) was designed to be used in conjunction with the (GAA)•(TTC) RP-PCRs. In the current study, the GAAGGA motif was identified in 12% (2/17) of individuals carrying alleles of ≥250 triplet repeats (i.e. ≥125 hexanucleotide repeat expansion). Unlike prior reports, no [(GAA)_m_(GCA)_n_]_z_ motifs were observed in our cohort. This motif is frequently found in Asian populations, but it is more rarely found in individuals of European descent. The [(GAA)_m_(GCA)_n_]_z_ expansion was identified in 1.4 and 5.3% of patients, with ≥250 repeat units, in a French-Canadian and German cohort, and a Chinese cohort, respectively.^1,19^

Our study has some limitations that need to be considered. First, the absence of quantitative data from ataxia rating scales, specifically the Scale for the Assessment and Rating of Ataxia (SARA). The lack of SARA scores hinders the assessment of disease severity and the comparison of its annual progression. Second, thus far only a portion of ataxia index patients were genetically explored by whole-exome sequencing. Therefore, it is likely that the current proportion of positive cases within the Cypriot LOCA cohort may have been underestimated. Furthermore, NCS data were available in only 5 out of 12 patients. While the ataxia cohort size of 155 index patients may appear limited, it is important to note that the current population estimate in the Republic of Cyprus is 923 000.^44^ Additionally, this study was carried out in the only referral centre for ataxias in the Republic of Cyprus, making the findings of this study representative of the entire population.

In conclusion, this report presents the first cases of SCA27B in the Cypriot population. Our study comprehensively compares different electrophoretic techniques and underscores the importance of a single reliable diagnostic test for accurate allele sizing. The *FGF14* GAA repeat expansion is the third such expansion associated with cerebellar ataxia to be identified in Cyprus, following the *FXN* GAA and *RFC1* AAGGG repeat expansions both causing autosomal recessive diseases.^26,27^ Currently, SCA27B represents the predominant aetiology of ADCA within the Cypriot population. Moreover, the identification of SCA27B as this population’s first dominant repeat expansion ataxia holds significant clinical value as no patients were ever identified in the Cyprus population to carry any of the relatively common polyglutamine SCAs. Finally, our data strongly advocate including *FGF14* GAA repeat expansion testing as a first-tier genetic diagnostic approach for patients presenting with LOCA.

## Supplementary Material

fcae479_Supplementary_Data

## Data Availability

The data generated and analysed during this study are available through any reasonable request from the corresponding author. Any personal data will not be available due to privacy and ethics restrictions.
