# Supplementary material for: The FGF14 GAA repeat expansion is a major cause of ataxia in the Cypriot population
Source: Brain Commun. 2025 Jan 3;7(1):fcae479. doi: 10.1093/braincomms/fcae479 (PMC11724429; doi:10.1093/braincomms/fcae479)
Supplement: fcae479_Supplementary_Data [file fcae479_supplementary_data.pdf]

Supplementary Figures and Tables

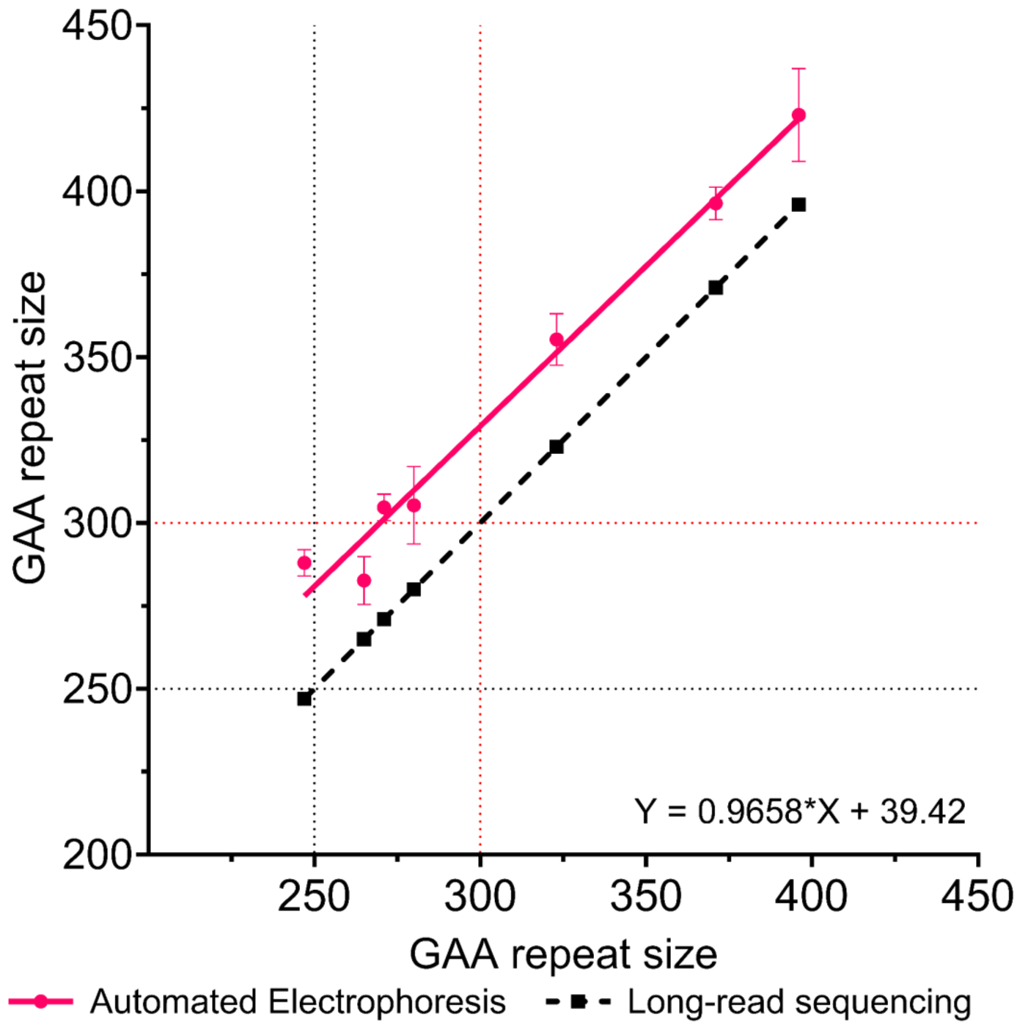

Supplementary Figure 1: Each data point represents the seven samples sized by Oxford nanopore long-read sequencing, which were employed as calibrators to adjust the Tapestation 4150 automated electrophoresis measurements.

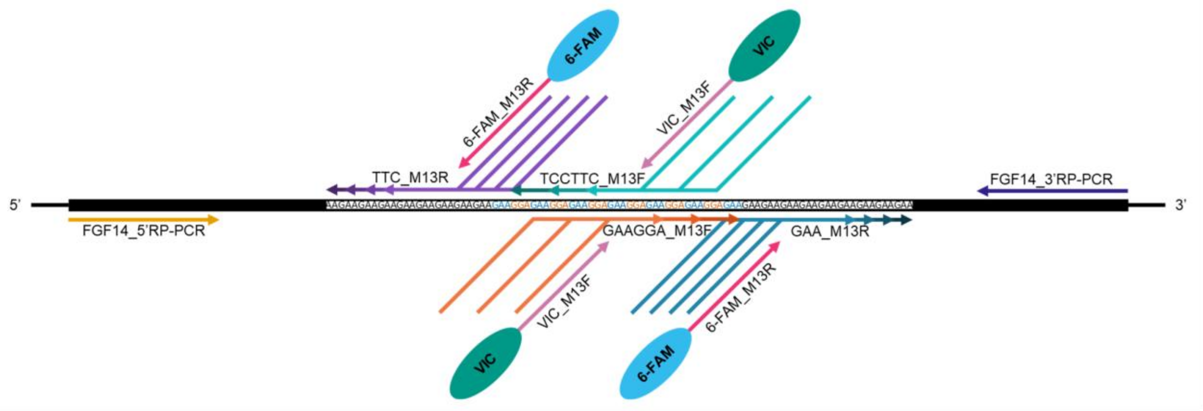

Supplementary Figure 2: Schematic representation of the primers designed for the four RP-PCR reactions targeting the GAA and the GAAGGA repeats in 5’ and 3’ directions

Reaction	Primer	Sequence	Conc.
5’ RP-PCR GAA	*FGF14*_5’RP-PCR	5'–AGCAATCGTCAGTCAGTGTAAGC–3’	0.5 µM
	TTC_M13R	5'–CAGGAAACAGCTATGACC–CTTCTTCTTCTTCTTCTTCTT–3’	0.2 µM
	6-FAM_M13R	5’–6-FAM–CAGGAAACAGCTATGACC–3’	0.5 µM
5’ RP-PCR GAAGGA	*FGF14*_5’RP-PCR	5'–AGCAATCGTCAGTCAGTGTAAGC–3’	0.5 µM
	TCCTTC_M13F(-20)	5'–GTAAAACGACGGCCAGTG–TCCTTCTCCTTCTCCTTCTCC–3’	0.2 µM
	VIC_M13F(-20)	5'–VIC–GTAAAACGACGGCCAGTG–3’	0.5 µM
3’ RP-PCR TTC	*FGF14*_3’RP-PCR	5’–TGCCCACATAGAGCTTAGTCT–3’	0.5 µM
	GAA_M13R	5'–CAGGAAACAGCTATGACC–GAAGAAGAAGAAGAAGAAGAA–3’	0.2 µM
	6-FAM_M13R	5’–6-FAM–CAGGAAACAGCTATGACC–3’	0.5 µM
3’ RP-PCR TCCTTC	*FGF14*_3’RP-PCR	5’–TGCCCACATAGAGCTTAGTCT–3’	0.5 µM
	GAAGGA_M13F(-20)	5'–GTAAAACGACGGCCAGTG–GAAGGAGAAGGAGAAGGAGAA–3’	0.2 µM
	VIC_M13F(-20)	5'–VIC–GTAAAACGACGGCCAGTG–3’	0.5 µM
Cycling conditions	98°C x 1 min<br><br>[98°C x 3 sec<br>60°C x 10 sec<br>72°C x 2 min] x 35 cycles		

Supplementary Table 1: Experimental conditions used for bidirectional (GAAGGA)•(TCCTTC) RP-PCR and modified bidirectional (GAA)•(TTC) RP-PCR. The primer sequences are colour-coded to match the primer illustrations above.

Patients	P1	P2[*1]	P3	P4	P5	P6	P7	P8	P9	P10	P11	P12
**$(GAA)^n$ size**	489	482	391	374	353	334	311	311	297	286	281	251
**Sex**	M	F	M	M	F	F	F	M	M	F	F	M
**Age at onset (years)**	52	61	50	58	64	69	65	70	76	25	71	59
**Age of first examination (years)**	55	63	65	68	69	79	67	77	79	28	81	65
**Gait ataxia**	+	+	+	+	+	+	+	+	+	+	+	+
**Upper limb ataxia**	+	+	+	+	+	-	-	+	+	-	+	+
**Truncal ataxia**	-	+	+	+	+	-	-	+	-	+	-	-
**Lower limb ataxia**	+	+	+	+	+	+	+	+	+	+	+	+
**Downbeat nystagmus**	-	+	-	-	-	-	-	-	+	+	+	+
**Gaze-evoked nystagmus**	-	+	-	-	+	-	-	+	+	+	+	+
**Dysphagia**	-	-	+	+	-	+	-	-	-	-	-	-
**Dysarthria**	-	+	+	+	+	+	+	-	-	-	-	+
**Episodic symptoms**	+	-	-	+	-	-	-	-	-	-	-	-
**Visual disturbances**	-	+	+	-	+	-	-	-	-	-	-	-
**Vertigo and/or dizziness**	-	-	-	-	+	-	-	-	+	-	+	-
**Alcohol intolerance**	-	-	-	-	-	-	-	-	-	-	-	+
**Exercise-triggered symptoms**	-	-	-	-	+	-	-	-	-	-	-	-
**Mobility Aid**	No	No	WC	WC	No	No	No	U/B	No	U/B	No	WC
**MRI findings**	VA	CA	VA	CA & WMLs	WMLs	WMLs	Normal	Normal	WMLs	Normal	CA	Global
**Nerve conduction studies**	Normal	Normal	N/A	N/A	Normal	N/A	Normal	N/A	N/A	Abnormal	N/A	N/A

Supplementary Table 2: M, male; F, female; WC, wheelchair-bound; U/B, unilateral or bilateral support; CA, cerebellar atrophy; VA, vermis atrophy; WMLs, white matter lesions; N/A, not available.
*[1] Russian ancestry.
